# Supplementary material for: Columba: fast approximate pattern matching with optimized search schemes
Source: Bioinformatics. 2025 Dec 4;41(12):btaf652. doi: 10.1093/bioinformatics/btaf652 (PMC12724072; doi:10.1093/bioinformatics/btaf652)
Supplement: btaf652_Supplementary_Data [file btaf652_supplementary_data.zip › Columba_Suppl_Data.pdf]

Columba: Fast Approximate Pattern Matching with Optimized Search Schemes
Supplementary Data

Luca Renders, Lore Depuydt, Travis Gagie, Jan Fostier

1 A Primer on Search Schemes

In lossless approximate pattern matching (APM), one needs to identify *all* approximate occurrences of a search pattern P (e.g., a read) within a text T (one or more reference genomes) with up to k errors (substitutions, insertions, or deletions). Formally, we wish to identify all substrings O of T such that $d_{\text{edit}}(O, P) \leq k$. Here, $d_{\text{edit}}(.,.)$ denotes the edit distance between two strings.

In order to understand search schemes, we first explain a backtracking algorithm, followed by the pigeonhole-based search. The focus is on explaining the underlying ideas; for technical details, we refer to the literature.

1.1 Backtracking

A backtracking algorithm provides a simple but inefficient solution to the APM problem. Given an index of T, such as the FM-index, candidate occurrences O^c are spelled out incrementally, character by character, in a depth-first manner. For simplicity, we assume candidate occurrences O^c are spelled left-to-right; the reasoning is analogous for indexes that spell right-to-left, such as the FM-Index. Superscript c refers to the fact that strings O^c are *candidate* occurrences, i.e., additional characters still have to be appended to O^c in order to become an actual occurrence O such that $d_{\text{edit}}(O, P) \leq k$.

The backtracking algorithm is illustrated in Fig. 1. We start with $O^c = \varepsilon$, the empty string. At each step, one character is appended to the current O^c string. The role of the FM-index is to ensure that only strings O^c are produced that exist in T. We keep track of the edit distance between O^c and (prefixes of) P using a banded dynamic programming matrix D. Each matrix element $D(i, j)$ (zero-based indexing) represents $d_{\text{edit}}(O^c[1, i], P[1, j])$, i.e., the edit distance between the first i characters of O^c and the first j characters of P. Because we are interested only in occurrences with at most k errors, a banded matrix with at most k elements left and k elements right of the diagonal suffices. At each step, one of these two (mutually exclusive) scenarios occurs:

1. All elements of row $|O^c|$ of matrix D exceed k, where $|O^c|$ denotes the length of string O^c. In other words, the edit distance between string O^c and any prefix of P already exceeds the maximum number of k errors. At this point, O^c should no longer be extended because any further extension will also exceed k errors (the edit distance can only increase, not decrease by further extending O^c). The algorithm backtracks to the most recent branching point with unexplored characters and continues the search from there. This case is illustrated in Fig. 1, top-left panel.

2. Not all elements of row $|O^c|$ of matrix D exceed k. String O^c will be further extended in the next step. Additionally, if matrix element $D(|O^c|, |P|) \leq k$, then O^c should be reported as an approximate occurrence of P. Note that O^c represents an index occurrence and that

O^c may have multiple repeated occurrences in T. In the case of the FM-index, the starting positions of all repeats of O^c in T are decoded using the suffix array. Fig. 1 illustrates some cases of reported occurrences.

Clearly, the backtracking algorithm exhaustively enumerates all approximate occurrences of P in T, and is therefore a solution to the APM problem. With an index that can perform character extensions in $\mathcal{O}(1)$ (constant) time (e.g. FM-index), and given that each character extension requires computing at most $2k+1$ cells in a row of D, the backtracking algorithm has a runtime that is proportional to the total number of strings O^c that are enumerated. This equals the number of nodes in the search tree induced by the backtracking algorithm (cf. the tree in Fig. 1). We also refer to this as the *search space*. The search space is constrained by two factors:

1. Strings O^c must exist in T. In other words, we do not exhaustively enumerate all possible strings as candidate occurrences, only the subset of strings that exist as a substring of T. This is the role of the index.

2. At each point, either $d_{\text{edit}}(O^c, P) \leq k$, or it must still be possible to further right-extend O^c to a longer string O' such that $d_{\text{edit}}(O', P) \leq k$. In other words, as soon as a string O^c differs too much from (a prefix of) P, all further extensions of O^c are skipped.

For mapping Illumina reads against, e.g., the human genome, the backtracking algorithm is computationally impractical beyond $k = 2$ errors. This is because the search tree is densely branched near its root (i.e., most nodes in the upper levels of the search tree have four outgoing edges corresponding to characters A, C, G, and T). The reason for this is that a) short strings O^c have a high probability of occurring in T; b) short strings O^c have likely not yet exceeded the maximum number of errors w.r.t. pattern P. In other words, the backtracking algorithm spends an excessive amount of time enumerating a combinatorially exploding number of short strings, only to find out that the vast majority of these sequences cannot be extended to become an approximate occurrence of P. Lam et al. were the first to highlight this issue [Lam et al., 2009]. To address this, they proposed a strategy based on the pigeonhole principle, explored in the next section.

1.2 Pigeonhole-based Search

Consider any approximate occurrence O of P with at most k errors under the edit distance. The pigeonhole principle implies that if we partition pattern P into $k+1$ non-overlapping parts, at least one part of P is exactly contained in O. It is easy to understand why. String P can be transformed into string O using at most k edit operations (insertions, deletions, or substitutions). If P is partitioned into $k+1$ parts, then these k distinct edit operations can be applied to at most k different parts of P, leaving at least one part of P unchanged.

For example, assume we are looking for occurrences of P with at most $k = 1$ error. By partitioning P into $k+1 = 2$ parts (i.e., $P = P_1 \circ P_2$, with $\circ$ the string concatenation operator), any occurrence O either has no errors at all or has a single error in either P_1 or in P_2. In all cases, O contains at least one error-free part of P. To identify all occurrences O of P, we conduct two *searches* S_1 and S_2. Each search identifies a subset of the approximate occurrences O of P. Search S_1 will identify all approximate occurrences O of P for which part P_1 is error-free. Similarly, search S_2 will identify all approximate occurrences O of P for which part P_2 is error-free. Collectively, both searches identify all occurrences O. Search S_1 is conducted as follows. First, by means of the index, part P_1 is matched using exact pattern matching. Next, this match (if it exists) is extended using the backtracking algorithm to also match P_2, allowing for at most one error. Similarly, in search S_2, part P_2 is first matched using exact pattern

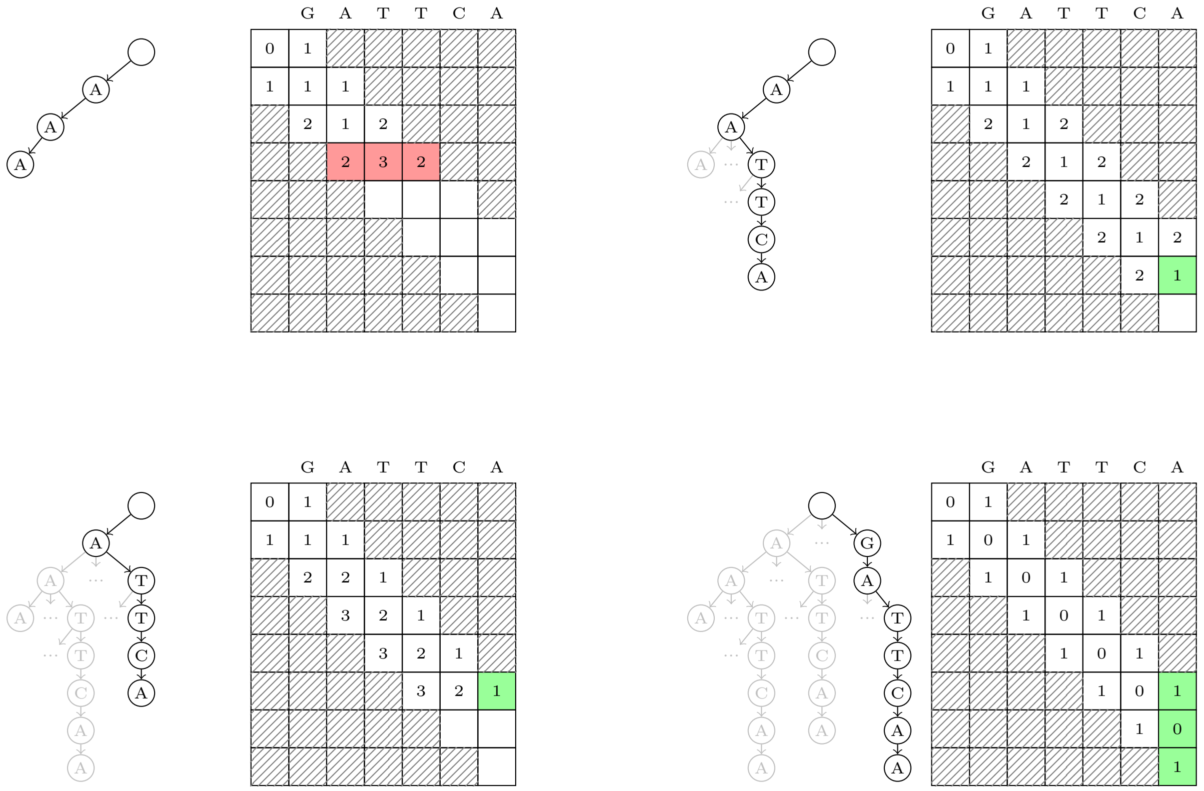

Figure 1: Example of the backtracking algorithm under the edit distance with P = GATTCA and a maximum of $k = 1$ errors. Cells shaded in green indicate that an approximate occurrence O was reported. **Top left**: during the first three steps, strings O^c = A, AA, and AAA, respectively, are enumerated. For string O^c =AAA, all elements on row $|O^c| = 3$ of matrix D (zero-based indexing) exceed $k = 1$ (shaded in red). Thus, all further extensions of AAA will also exceed k errors and can therefore be skipped. The algorithm backtracks to explore alternative extensions of AA. Note that rows of matrix D corresponding to the string O^c = AA do not have to be recomputed. **Top right**: string O = AATTCA is reported as an approximate occurrence of P at edit distance 1 (green shaded cell in D). **Bottom left**: a second approximate occurrence O = ATTCA is reported at edit distance 1. **Bottom right**: string O = GATTCAA is reported at edit distance 1; in the previous step, GATTCA was reported at edit distance 0; in the step before that, GATTC was reported at edit distance 1 (cf. cells shaded in green).

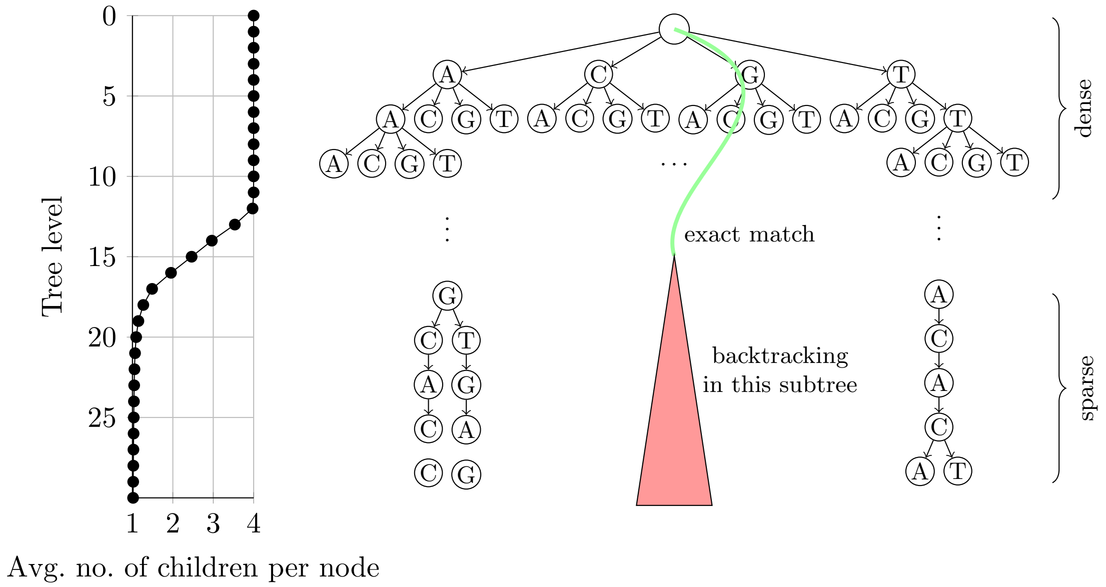

Figure 2: Left graph: the average number of children per node as a function of tree level (data for the human reference genome GRCh38). Upper levels are densely branched (most nodes have four children), whereas lower levels are sparsely branched (most nodes have a single child). Right: corresponding search tree. By leveraging the pigeonhole principle, an initial exact match of a part of P bypasses the densely branched region near the root of the search tree. Backtracking is performed only in lower, sparsely branched levels of the search tree.

matching. Next, this match is extended (to the left) using the backtracking algorithm, thus also matching part P_1 with a single error allowed.

Leveraging the pigeonhole principle yields significant computational gains (see Fig. 2). The initial exact pattern matching conducted during both searches bypasses the upper levels of the search tree, which are densely branched (i.e., most nodes contain four children: A, C, G, and T). Backtracking has to be performed only in a subtree that is much more sparsely branched (i.e., most nodes only have a single child). Therefore, exploring this sparse subtree is typically much faster.

The pigeonhole principle naturally generalizes to an arbitrary number of errors. If k errors are allowed, the pattern P is partitioned into $k+1$ parts, and $k+1$ searches S_i ($i = 1, \ldots, k+1$) are defined. Each search S_i identifies the subset of approximate occurrences of P that contain part P_i without any errors. This part P_i is matched first, with no errors allowed. The match is then extended to the left using backtracking, thereby matching parts $P_{i-1}, \ldots, P_1$, allowing up to k errors. Finally, the remaining parts $P_{i+1}, \ldots, P_{k+1}$ are matched by extending to the right, again using backtracking and cumulatively allowing up to k errors.

A *bidirectional* index, such as the bidirectional FM-index [Lam et al., 2009], is required to support extending partial matches in both directions. Together, all searches S_i ($i = 1, \ldots, k+1$) identify all approximate occurrences of P, thereby solving the approximate pattern matching (APM) problem. However, some occurrences may be identified by multiple searches. For example, an occurrence with more than one error-free part may be identified multiple times. Such redundant occurrences must be filtered in a post-processing step (see further).

In practice, pigeonhole-based search significantly outperforms naive backtracking. For example, Renders [2020] reports a runtime of 22.4 seconds to map 100 000 Illumina reads (250 bp) with up to $k = 4$ errors to the human reference genome using pigeonhole-based search, whereas backtracking takes more than 68 hours (!) for the same task (see Table 7.1 in [Renders, 2020]).

1.3 Search Schemes

Search schemes [Kucherov et al., 2016] provide a flexible framework in which various search strategies can be expressed. The goal is again to identify all approximate occurrences of a pattern P, allowing for at most k errors. The pattern P is partitioned into p non-overlapping parts. In practice, the most efficient search schemes typically use either $p = k + 1$ parts (as in pigeonhole-based search) or $p = k + 2$ parts. A search scheme $\mathcal{S}$ is defined as a collection of $|\mathcal{S}|$ searches S_i, i.e., $\mathcal{S} = \{S_i\}$, with $i = 1, \ldots, |\mathcal{S}|$.

The following example shows two such schemes: $\mathcal{S}_{\text{ph}}$, which expresses the pigeonhole-based search from Section 1.2, and $\mathcal{S}_{\text{MinU}}$, a more efficient search scheme. Both solve the approximate pattern matching (APM) problem for up to $k = 4$ errors.

$$\begin{array}{lcc} & \mathcal{S}_{\text{ph}} & \mathcal{S}_{\text{MinU}} \\ \hline S_1 & (12345,\ 00000,\ 04444) & (12345,\ 00222,\ 02244) \\ S_2 & (21345,\ 00000,\ 04444) & (23145,\ 00000,\ 01244) \\ S_3 & (32145,\ 00000,\ 04444) & (32145,\ 01111,\ 01244) \\ S_4 & (43215,\ 00000,\ 04444) & (45321,\ 00003,\ 01444) \\ S_5 & (\underbrace{54321}_{\pi},\ \underbrace{00000}_{L},\ \underbrace{04444}_{U}) & (\underbrace{54321}_{\pi},\ \underbrace{01114}_{L},\ \underbrace{01444}_{U}) \end{array} \tag{1}$$

Both search schemes have $|\mathcal{S}| = 5$ searches S_i $(i = 1, \ldots, 5)$. Each search S_i consists of a triplet (π, L, U) of arrays of exactly p numbers, with p the number of parts of P. For ease of notation, the arrays π, L, and U are written without separator symbols between their elements. The π array is a permutation of values $\{1, 2, \ldots, p\}$ that specifies the order in which the p parts of P have to be matched. For example, search S_3 of $\mathcal{S}_{\text{MinU}}$, illustrated in Fig. 3, has $\pi = 32145$. This means that part P_3 is matched first, followed by parts P_2, P_1, P_4, and P_5, in that order. In general, the parts are matched in the order $P_{\pi[1]}, P_{\pi[2]}, \ldots, P_{\pi[p]}$. Similarly, L and U are arrays of length p. Specifically, values $L[i]$ and $U[i]$ denote, respectively, the lower and upper bound to the cumulative number of errors after part $P_{\pi[i]}$ has been matched.

Search scheme $\mathcal{S}_{\text{ph}}$ corresponds to the pigeonhole-based search: during each search S_i, part P_i is first matched without errors, followed by a left-extension with parts $P_{i-1}, \ldots, P_1$ and a right-extension with parts $P_{i+1}, \ldots, P_p$. These extensions are performed using backtracking, allowing for at most k errors.

By comparing $\mathcal{S}_{\text{ph}}$ and $\mathcal{S}_{\text{MinU}}$, it is easy to see that $\mathcal{S}_{\text{MinU}}$ is more efficient. Whereas $\mathcal{S}_{\text{ph}}$ immediately allows for the maximum of $k = 4$ errors when matching part $P_{\pi[2]}$, the allowed number of errors in the searches of $\mathcal{S}_{\text{MinU}}$ increases only gradually as more parts are added. Additionally, $\mathcal{S}_{\text{MinU}}$ also imposes a non-zero lower bound on the number of errors. Branches that do not satisfy this lower bound can be discarded during backtracking, thus reducing the size of the search tree and hence the runtime.

Nevertheless, it should be emphasized that, collectively, all searches of $\mathcal{S}_{\text{MinU}}$ identify all approximate occurrences O of P with up to $k = 4$ errors. The search scheme is said to be *lossless*. Formally, a search scheme is lossless if it *covers* all possible *error configurations*. An error configuration $(e_1, \ldots, e_p)$ is a distribution of at most k errors over the p parts of P, i.e., $\sum_{i=1}^{p} e_i \leq k$. For example, $(0, 2, 0, 2, 0)$ is an error configuration where parts P_1, P_3, and P_5 contain no errors, and parts P_2 and P_4 each contain two errors. A search S *covers* an error configuration if $L[i] \leq \sum_{j \leq i} e_{\pi[j]} \leq U[i]$ for all $i = 1 \ldots p$. A search scheme is called *lossless* if each error configuration is covered by at least one of its searches. Note that there are $\sum_{i=0}^{k} \binom{p+i-1}{p-1} = \binom{p+k}{k}$ possible ways to distribute up to k errors over p parts. In practice, for larger values of k, checking if a search scheme is lossless is done by a computer program.

There have been several efforts in literature to design efficient search schemes. We use the $\mathcal{S}_{\text{MinU}}$ search schemes, which have been designed for up to $k = 13$ errors. These search

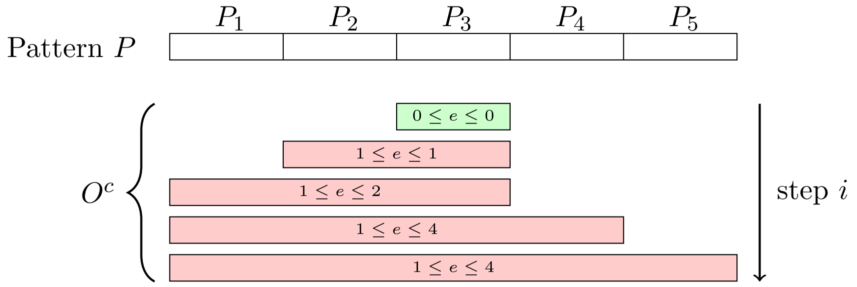

Figure 3: Visualization of search $S_3 = (32145, 01111, 01244)$ of search scheme $\mathcal{S}_{\text{MinU}}$ for $k = 4$ errors. Pattern P is partitioned into $p = k + 1 = 5$ parts. First, part P_3 is matched exactly (shaded in green). Next, this match is left-extended with P_2 and P_1, followed by a right-extension with P_4 and P_5 using the backtracking algorithm (shaded in red). After each step i, candidate occurrence O^c should contain a cumulative number of errors $L[i] \le e \le U[i]$.

schemes outperform pigeonhole-based search by a significant margin. All $\mathcal{S}_{\text{MinU}}$ search schemes are lossless.

1.4 Filtering Redundant Occurrences

Columba is designed to exhaustively report occurrences; however, it does not report *redundant* occurrences. Redundant occurrences refer to either the same occurrence being reported multiple times, or to slightly different representations of what is essentially the same underlying occurrence in T. Redundant occurrences offer no additional biological insights and thus should be filtered from the output.

First, we consider the case of the same occurrence in T being reported multiple times. This occurs when a certain error configuration is covered by multiple searches in a search scheme. For example, using the pigeonhole-based search scheme $\mathcal{S}_{\text{ph}}$, an exact occurrence of P will be reported k times, i.e., once by each search. Even when each error configuration is covered only by a single search, the same occurrence may be reported by multiple searches. For example, consider following search scheme for $k = 1$ errors and $p = 2$ parts:

$$\begin{aligned} S_1 &= (12,\ 00,\ 01) \\ S_2 &= (21,\ 01,\ 01) \end{aligned} \tag{2}$$

Search S_1 covers error configurations $(0, 0)$ and $(0, 1)$, whereas S_2 covers $(1, 0)$, hence the searches cover disjoint sets of error configurations. However, the same occurrence may be associated with multiple error configurations when it has insertions at the partition boundaries. For example, consider P = GATCAT, with P_1 = GAT and P_2 = CAT. Then O = GATACAT will be redundantly reported by both searches S_1 and S_2, because the inserted character can be assigned to either P_2 (left below case) or P_1 (right below case).

G	A	T	-	C	A	T
G	A	T	A	C	A	T

G	A	T	-	C	A	T
G	A	T	A	C	A	T

Multiply-reported occurrences are easily filtered in a post-processing step.

Next, we consider the case of near-identical occurrences. This refers to occurrences in the reference (pan-)genome that overlap substantially, but have slightly different start and/or end coordinates in T. Consider, for example, an exact occurrence O of P and assume that up to k errors are allowed. Without redundancy filtering, there would be $\sum_{i=1}^{k} 4i = 2k(k+1)$ redundant occurrences reported at a higher edit distance, simply by adding or removing leading and/or trailing characters in O. Clearly, the problem exacerbates with increasing k. For example, for

$k = 1$, an exact occurrence of P = GATCAT at coordinates $[c, c+6)$ (where $[., .)$ denotes a half-open interval) in T could also be redundantly reported at coordinates $[c+1, c+6)$, $[c-1, c+6)$, $[c, c+5)$, and $[c, c+7)$, all at edit distance 1.

T	...	A	G	A	T	C	A	T	A	...
O_1			G	A	T	C	A	T		
O_2		A	G	A	T	C	A	T		
O_3			G	A	T	C	A	T	A	
O_4				A	T	C	A	T		
O_5			G	A	T	C	A			

Columba will report only O_1 at edit distance 0 at this locus in T, and overlapping occurrences $O_2, \ldots, O_5$ at edit distance 1 are considered redundant. Columba considers occurrences to be overlapping if their start coordinates differ by fewer than $2k + 1$ characters. Among a set of overlapping occurrences, Columba reports only the occurrence with the lowest edit distance as the representative occurrence, and considers overlapping occurrences (with the same or higher edit distance) as redundant.

For example, consider the following low-complexity sequence region in T and P = ATATA:

T	...	A	T	A	T	A	T	A	T	A	T	A	T	A	...
O_1		A	T	A	T	A									
O_2				A	T	A	T	A							
O_3						A	T	A	T	A					
O_4								A	T	A	T	A			
O_5										A	T	A	T	A	

When $k = 0$ is used, Columba will report all exact occurrences $O_1, \ldots, O_5$, because at $k = 0$, they are considered to be non-overlapping. When $k = 1$ is used, only occurrences O_1, O_3, and O_5 will be reported because occurrence O_2 and O_4 overlap with, for example, O_3 and are thus considered redundant. Note that when $k = 1$, each exact occurrence O_i would also have four additional occurrences at edit distance 1, which are also filtered. Note that different lossless tools may use different strategies to filter redundant occurrences. For example, one lossless tool might report O_2 and O_4, whereas Columba would report O_1, O_3, and O_5.

1.5 Dynamic Partitioning of Search Patterns

Search schemes do not require the search pattern P to be partitioned into equal-length parts. For a search scheme to be lossless, it suffices that all searches collectively cover all error configurations. The length of each part is irrelevant, as long as the same part lengths are consistently used across all searches of the search scheme. Depending on the sequence content of P, non-uniform part sizes may be computationally beneficial. For example, consider again the simple search scheme for $k = 1$ errors and $p = 2$ parts from equation 2. Now assume that part P_1 occurs (significantly) more often in T than P_2, for example, because P_1 is a low-complexity sequence whereas P_2 is not. This means search S_1 would likely require more work than search S_2, because backtracking has to be performed in a larger subtree. We hereby assume that the number of occurrences of P_1 is a good proxy for the size of the underlying subtree. By increasing the size of P_1 (and thus decreasing the size of P_2), workload can be shifted from S_1 to S_2. The decrease in workload in S_1 can be much larger than the increase in workload in S_2, and therefore, there is a net performance gain. In their seminal work, Kucherov et al. [2016] provide a mathematical foundation for this observation. The concept is illustrated in Fig. 4.

Renders et al. [2021] propose a simple heuristic algorithm to determine good part sizes of P. Note that this algorithm is applied to each pattern P individually, and that different part sizes may be used for different patterns P, hence the name *dynamic* partitioning. However,

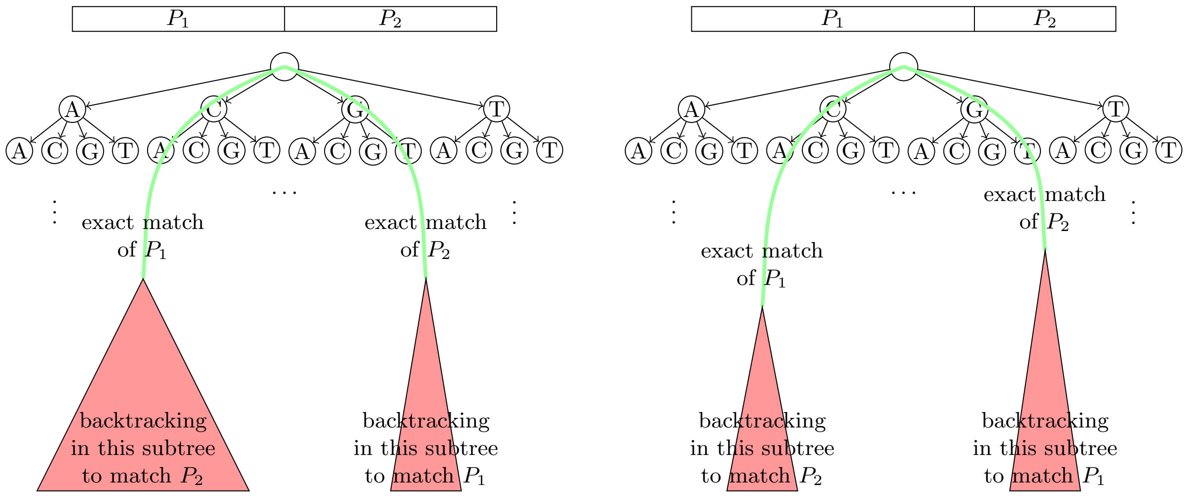

Figure 4: Left: pattern P is partitioned into equal-length parts. Because P_1 has more exact occurrences in T than P_2, the subtree to match P_2 during search S_1 is larger than the subtree to match P_1 during search S_2. Right: by extending P_1 (and shortening P_2), the workload is more evenly distributed across searches. This often results in a net reduction of search space.

we emphasize that once part sizes are determined for a specific pattern, these part sizes are consistently used across all searches of the search scheme to ensure its lossless character. Note that the heuristic must be very (computationally) lightweight in order not to incur much overhead. Even though the heuristic offers little guarantee for one individual pattern, it is shown to provide good performance *on average*, across a large number of patterns P.

1.6 Dynamic Selection of Search Schemes

It is often possible to define multiple 'equally good' search schemes for given values of k and p. For example, by mirroring the part numbering, an equivalent search scheme is obtained. For example, for $k = 4$ errors and $p = k + 1 = 5$ parts, the three search schemes below are equivalent.

	$\mathcal{S}_{\text{MinU,A}}$	$\mathcal{S}_{\text{MinU,B}}$	$\mathcal{S}_{\text{MinU,C}}$
S_1	(12345, 00222, **02244**)	(12345, 01114, 01444)	(12345, 01114, 01444)
S_2	(23145, 00000, 01244)	(21345, 00003, 01444)	(21345, 00003, 01444)
S_3	(32145, 01111, 01244)	(34521, 01111, **02244**)	(34521, 01111, 01244)
S_4	(45321, 00003, 01444)	(43521, 00000, 01244)	(43521, 00000, 01244)
S_5	(54321, 01114, 01444)	(54321, 00222, 01244)	(54321, 00222, **02244**)

(3)

This means that, across a large number of search patterns, the total expected workload is the same, regardless of the exact search scheme that is used. However, for an individual pattern P, this is not necessarily the case. To see this, first note that the expected workload associated with the different searches within each search scheme is not the same. For example, for $\mathcal{S}_{\text{MinU,A}}$, search S_1 (in bold) likely requires more work than the other searches in that search scheme, because S_1 allows for $U[2] = 2$ errors whereas the other searches allow only for a single error in their second part ($U[2] = 1$). Similarly, for $\mathcal{S}_{\text{MinU,B}}$, search S_3 has the highest expected workload and for $\mathcal{S}_{\text{MinU,C}}$, search S_5 has the highest expected workload. Note that these three searches perform their initial exact match at part P_1, P_3, and P_5, respectively.

This leads to the following heuristic: compare, for a given pattern P, the number of exact matches for P_1, P_3, and P_5. If P_1 has the fewest matches, execute search scheme $\mathcal{S}_{\text{MinU,A}}$; if P_3

has the fewest matches, execute search scheme $\mathcal{S}_{\text{MinU,B}}$; otherwise, execute $\mathcal{S}_{\text{MinU,C}}$. Again, this heuristic offers no strong guarantees for an individual pattern; however, it yields a substantial performance gain across a large number of patterns, on average (see Renders et al. [2024], Table 2). Because different search schemes may be selected for different patterns P, this technique is called *dynamic selection of search schemes*. Again, because each search scheme is lossless, the use of this heuristic maintains the lossless character.

1.7 Other Performance Optimizations

We briefly list other optimizations that improve the performance of search schemes. We refer to the literature for details. All of these performance optimizations maintain the lossless character of search schemes.

- **Design of search schemes**: Well-designed search schemes are imperative to good performance. Good lossless search schemes have been designed by Kucherov et al. [2016], Kianfar et al. [2017], Pockrandt [2019], and Renders et al. [2024]. We use the $\mathcal{S}_{\text{MinU}}$ search schemes designed by Renders et al. [2024] for up to $k = 13$ errors.

- **Optimized index**: For the FM-index, we use the $\mathcal{O}(1)$ bidirectional character extension as proposed by Pockrandt et al. [2017]. Rank operations on bitvectors are implemented using the fast rank9 algorithm by Vigna [2008]. To reduce cache-misses in the context of the backtracking algorithm, the bitvectors are memory-interleaved as proposed by Renders et al. [2021]. For the run-length compressed version of Columba, we use the cache-friendly bidirectional move structure [Depuydt et al., 2024].

- **In-text verification**: As explained in Section 1.1, (candidate) occurrences O^c are enumerated character by character using backtracking. Each character extension requires expensive random memory access. The FM-index provides information on the number of occurrences O^c in T. If this number of occurrences is small, rather than further extending O^c character by character using the FM-index, the locations of O^c in T are decoded using the suffix array and, for each location, the presence of an approximate occurrence of P at that location in T is checked using dynamic programming. This procedure can be computationally faster and maintains the lossless character of search schemes. This technique is detailed in Renders et al. [2022]. Note that this optimization applies only to the FM-index, not to the run-length compressed move structure, as in the latter, the text T is not stored in memory.

- **Bit-parallel implementation**: During backtracking, for each character extension, a total number of up to $2k + 1$ cells have to be computed on a row of the edit distance matrix D. Using a modified version of the bit-parallel algorithm by Hyyrö [2003], it is possible to compute an entire row in just a handful of CPU instructions. Similarly, the in-text verification is performed using a fast, bit-parallel algorithm. This technique is explained in more detail in Renders et al. [2022].

- **Avoiding redundant occurrences**: As explained in Section 1.4, redundant occurrences are filtered in a post-processing step. However, for performance reasons, it is beneficial to avoid reporting redundant occurrences already during backtracking. This avoids having to decode all their starting positions using the suffix array, only to find out that they overlap better, or equally good occurrences in T. For example, in the bottom-right panel of Fig. 1, occurrences $O =$ GATTCAA and GATTC at edit distance one should not be reported as they are redundant with $O =$ GATTCA at edit distance zero. Indeed, every occurrence of GATTCAA in T will overlap with GATTCA. Similarly, every occurrence of GATTC will overlap with GATTCA because GATTC has a unique right-extension, namely character

A (see Fig. 1, bottom-right panel). Filtering redundant occurrences during backtracking also plays an important role in the context of search schemes where P is matched part by part. After part $P_{\pi[i]}$ has been matched, in principle, a new alignment procedure should be started for each individual partial occurrence to extend it with part $P_{\pi[i+1]}$. However, some of these partial occurrences may be redundant. In Renders et al. [2021], the theory is developed on how to identify such redundant occurrences in between parts. This technique is important to maintain good performance in search schemes.

References

L. Depuydt, L. Renders, S. Van de Vyver, L. Veys, T. Gagie, and J. Fostier. b-move: Faster Bidirectional Character Extensions in a Run-Length Compressed Index. In *24th International Workshop on Algorithms in Bioinformatics (WABI 2024)*, volume 312, pages 10:1–10:18, Aug. 2024.

H. Hyyrö. A bit-vector algorithm for computing Levenshtein and Damerau edit distances. *Nord. J. Comput.*, 10(1):29–39, 2003.

K. Kianfar, C. Pockrandt, B. Torkamandi, H. Luo, and K. Reinert. FAMOUS: Fast approximate string matching using optimum search schemes. *CoRR*, 2017.

G. Kucherov, K. Salikhov, and D. Tsur. Approximate string matching using a bidirectional index. *Theoretical Computer Science*, 638:145–158, 2016.

T. Lam, R. Li, A. Tam, S. Wong, E. Wu, and S. Yiu. High throughput short read alignment via bi-directional BWT. In *IEEE International Conference on Bioinformatics and Biomedicine*, pages 31 – 36, Dec 2009.

C. Pockrandt, M. Ehrhardt, and K. Reinert. EPR-Dictionaries: A Practical and Fast Data Structure for Constant Time Searches in Unidirectional and Bidirectional FM Indices. In *Research in Computational Molecular Biology*, pages 190–206, 2017.

C. M. Pockrandt. *Approximate String Matching: Improving Data Structures and Algorithms.* Dissertation, Freie Universität Berlin, 2019.

L. Renders. Approximate sequence alignment using the bidirectional FM-index. Master's dissertation, Ghent University, 2020.

L. Renders, K. Marchal, and J. Fostier. Dynamic partitioning of search patterns for approximate pattern matching using search schemes. *iScience*, 24(7):102687, 2021.

L. Renders, L. Depuydt, and J. Fostier. Approximate pattern matching using search schemes and in-text verification. In *Bioinformatics and Biomedical Engineering (IWBBIO 2022)*, page 419–435, 2022.

L. Renders, L. Depuydt, S. Rahmann, and J. Fostier. Lossless approximate pattern matching: Automated design of efficient search schemes. *Journal of Computational Biology*, 31(10): 975–989, 2024.

S. Vigna. Broadword Implementation of Rank/Select Queries. In C. C. McGeoch, editor, *Experimental Algorithms*, pages 154–168, Berlin, Heidelberg, 2008. Springer Berlin Heidelberg. ISBN 978-3-540-68552-4. doi: 10.1007/978-3-540-68552-4_12.

2 Commands and Versions Used for the Different Tools

Table 1: Commands (except I/O) used in the alignment benchmarks. E stands for the maximal error rate and L stands for the average length of the reads (151 bp for the benchmarks on the human reference genome and 150 bp for the benchmarks on the bacterial pan-genome.

**Tool**	**Version**	**Command**
Yara	1.0.3	`./yara_mapper -y full -t 1 -e [E] -sa record`
RazerS3	3.4	`./razers3 -i [100 - E] -m 99999 -dr 0`
BWA-aln	0.7.19	`./bwa aln -N -n` [$1 - \sum_{i=0}^{\lfloor \frac{L \cdot E}{100} \rfloor - 1} \frac{e^{-\lambda} \cdot \lambda^i}{i!}$], with $e = 0.02$, or `./bwa aln -N -n 0`, if $E = 0$, followed by `./bwa samse`
Bowtie	1.3.1	`./bowtie -a --best --strata -v [E·L]`
Columba	2.0.2	`./columba -I [100-E] [--activate-CIGAR in RLC mode]`
BWA-MEM	0.7.19	`./bwa mem -L 10000`
BWA-MEM2	2.2.1	`./bwa mem -L 10000`
Bowtie2	2.5.4	`./bowtie2`
Bowtie2 VS	2.5.4	`./bowtie2 --very-sensitive`
Ropebwt3	3.9	`./ropebwt3 sw -t1`

3 Number of Occurrences per Read for Different Aligners

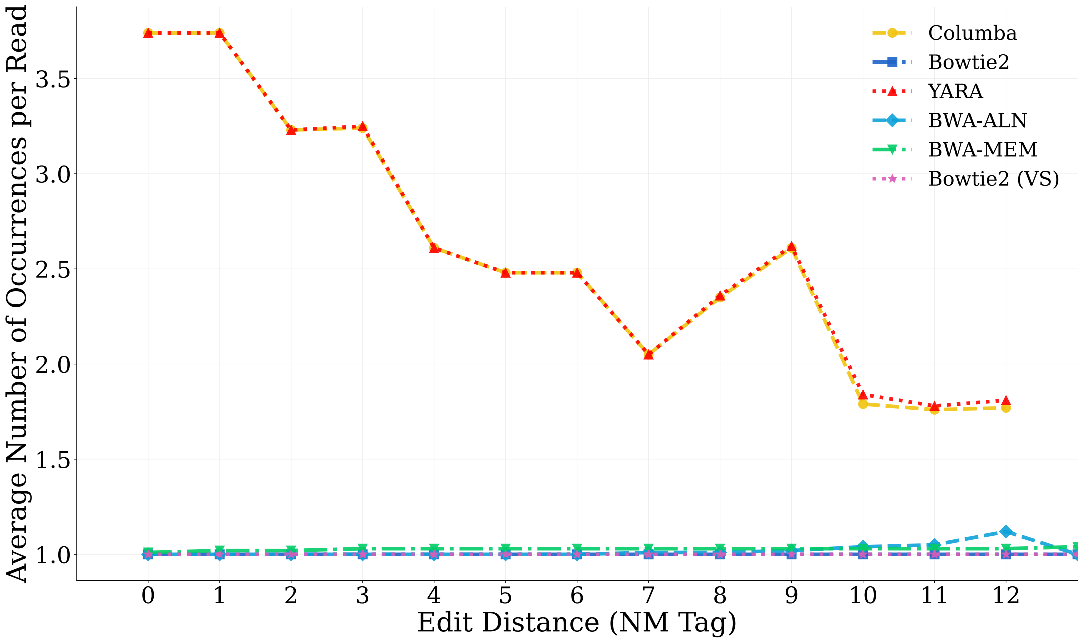

Figure 5: The average number of occurrences per read as a function of the edit distance for the different tools. Results are for the alignment of 1 million reads (151 bp in length) against the human reference genome, allowing a maximum error rate of 8%, i.e., at most $k = 12$ errors. Note that only a relatively small fraction of the reads has multiple occurrences: the median number of occurrences is 1 for all tools and all edit distances.

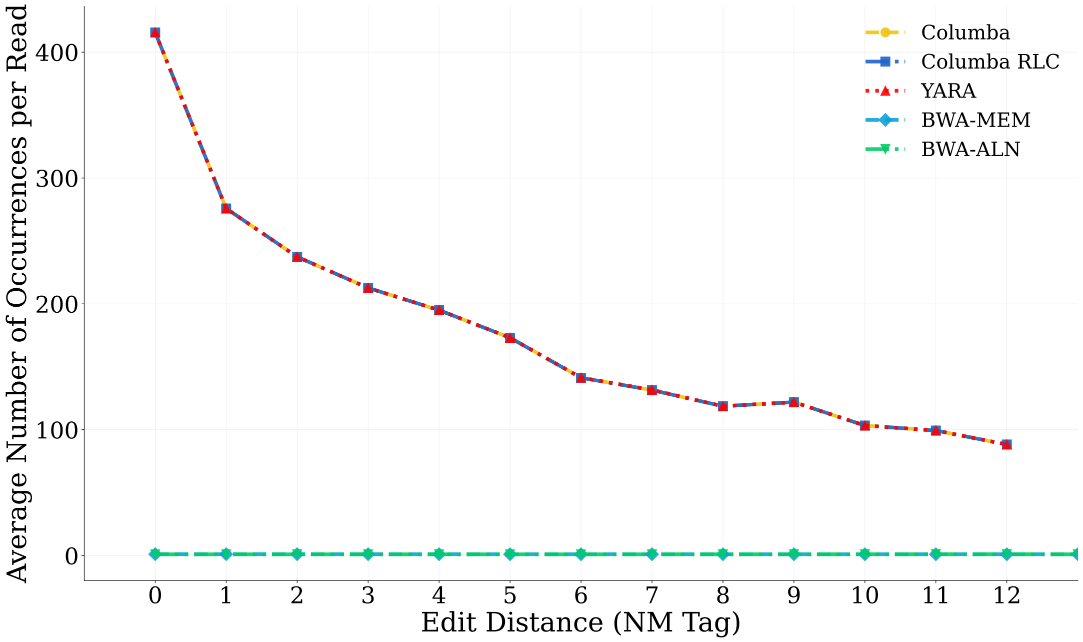

Figure 6: The average number of occurrences per read as a function of the edit distance for the different tools. Results are for the alignment of 1 million reads (150 bp in length) against the bacterial pan-genome, allowing a maximum error rate of 8%, i.e., at most $k = 12$ errors.

4 Analysis of the Output of Different Aligners

We provide a detailed analysis of the differences in SAM output between Columba and both Yara (lossless) and BWA-MEM(2) (lossy). For each input read, we first assess whether both aligners report the same (optimal) edit distance. If so, we then compare whether they report the same underlying occurrence(s) in the reference (pan-)genome for this optimal edit distance. Note that a read may have multiple occurrences. Since the lossless tools Yara and Columba were run in all-best mode, each of these occurrences shares the same (optimal) edit distance. We refer to such occurrences as co-optimal. As explained in Section 1.4, we define a tolerance threshold of $2k + 1$, where k is the maximum number of allowed errors. Occurrences reported by different tools are considered equivalent if their alignment positions in the reference differ by at most $2k + 1$ bp.

4.1 Columba versus Yara

Fig. 7 shows a detailed comparison of the SAM output between Columba and Yara for the alignment of 1 million reads (151 bp in length) against the human reference genome, allowing a maximum error rate of 8%, i.e., at most $k = 12$ errors.

- Both tools produce identical output for $> 99.99\%$ of the reads.
- For 15 reads, Columba reports more occurrences at the optimal edit distance than Yara. For 7 of these, the discrepancy occurs because Columba—like BWA-MEM(2)—replaces non-ACGT characters (e.g., the 'N' character) in the reference (pan-)genome with a randomly chosen A, C, G, or T. Such substitution can yield an extra occurrence for a read. For the remaining 8 reads, Yara fails to report at least one co-optimal occurrence, for unknown reasons.
- For 4 reads, Yara does not report any occurrence whereas Columba does. All such occurrences overlap a non-ACGT character in the reference that was randomly replaced by a A, C, G, or T by Columba.
- For 27 reads, Columba reports occurrences with a better (i.e., lower) edit distance than Yara. This occurs again exclusively due to the replacement of non-ACGT characters.
- For 15 reads, Yara reports more occurrences than Columba. In all cases, this is because Columba considers these additional occurrences redundant—since their alignment positions differ by at most $2k + 1 = 25$ bp from other reported occurrences—whereas Yara does not.

Similarly, Fig. 8 shows a detailed comparison of the SAM output between Columba and Yara for the alignment of 1 million reads (150 bp in length) against the bacterial (pan-)genome, allowing a maximum error rate of 8%, i.e., at most $k = 12$ errors. For this dataset, all differences in output can be attributed to the different treatment of non-ACGT characters. Notably, Yara did not miss any alignments, and no discrepancies arose from the handling of redundant occurrences. The latter is likely because bacterial genomes, unlike the human reference genome, do not contain highly repetitive, low-complexity regions that lead to overlapping (redundant) occurrences.

We conclude that Yara and Columba produce near-identical output and that minor differences are caused by implementation details.

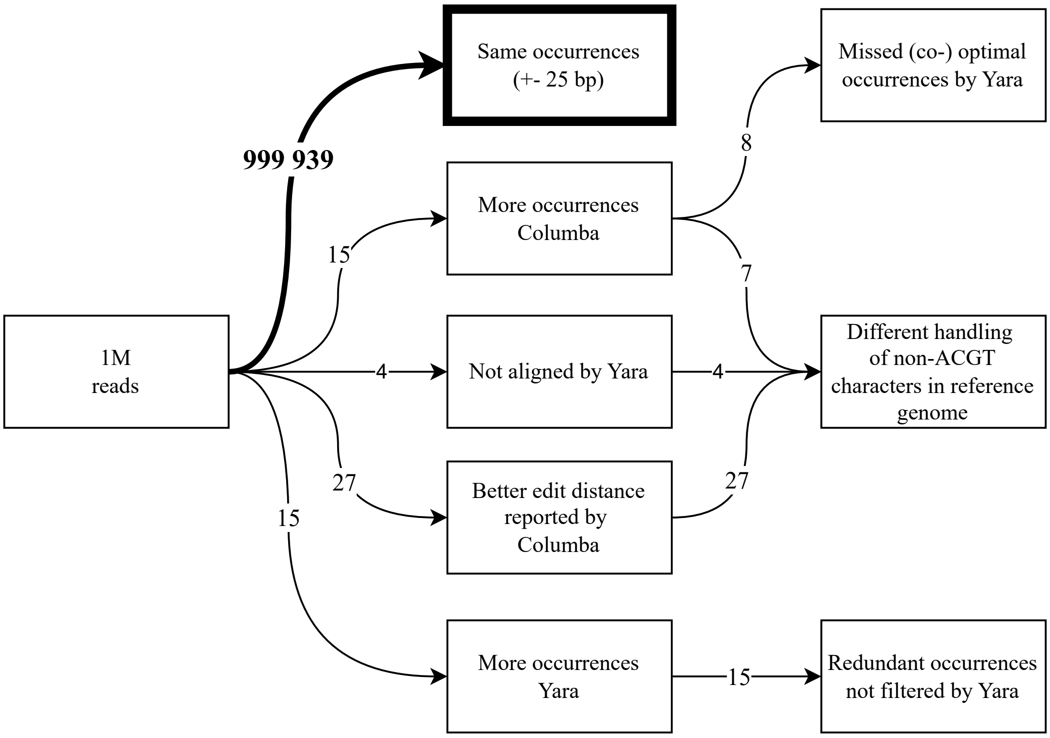

Figure 7: Comparison of the output between Columba and Yara for the alignment of 1 million reads (150 bp) against the human reference genome, allowing a maximum error rate of 8%.

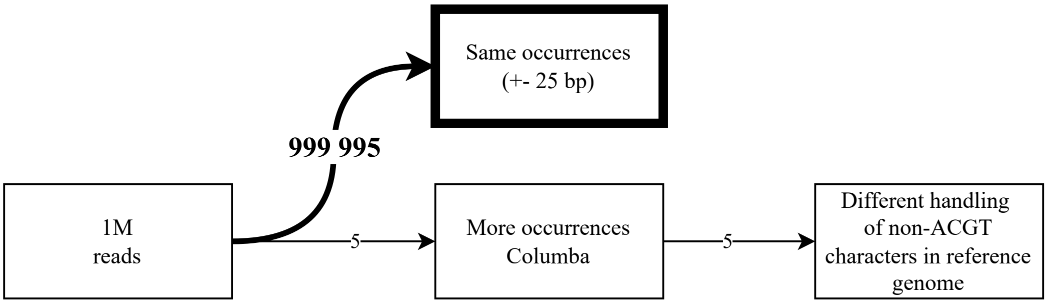

Figure 8: Comparison of the output between Columba and Yara for the alignment of 1 million reads (150 bp) against the bacterial (pan-)genome, allowing a maximum error rate of 8%.

4.2 Columba versus BWA-MEM(2)

Fig. 9 shows a detailed comparison of the SAM output between Columba and BWA-MEM2 for the alignment of 1 million reads (151 bp in length) against the human reference genome, allowing a maximum error rate of 8%, i.e., at most $k = 12$ errors.

- Both tools produce identical output for more than 95.35% of the reads.
- For 8 reads, at least one occurrence was reported by Columba but not by BWA-MEM2. We speculate that BWA-MEM2 failed to find a sufficiently long maximal exact match (MEM) between the reads and those occurrences.
- For 20 822 reads, Columba reports more occurrences at the optimal edit distance than BWA-MEM2. The vast majority of these occurrences are missed by BWA-MEM2. Few cases are due to non-ACGT characters in the reference.
- For 4 623 reads, Columba reports one or more occurrences with a better (i.e., lower) edit distance. In many of these cases (3 171 reads), BWA-MEM2 reports an overlapping alignment (within $2k + 1 = 25$ bp), but at a higher edit distance. For 1 443 reads, the optimal occurrence is not reported by BWA-MEM2 at all.
- For 2 reads, BWA-MEM2 reports more occurrences than Columba. This is either due to the presence of non-ACGT characters in the reference (1 read) or because BWA-MEM2 clipped the read, discarding part of it from the alignment (1 read).
- For the same two reasons, BWA-MEM2 reports one or more occurrences with a lower edit distance than Columba for 63 reads.
- For 20 968 reads, at least one occurrence was reported by BWA-MEM2 but not by Columba. In all cases, this is because either the error rate exceeds the maximum threshold of 8% (12 errors), or BWA-MEM2 applied read clipping, thereby discarding part of the read from the alignment.

Similarly, Fig. 10 shows a detailed comparison of the SAM output between Columba and BWA-MEM for the alignment of 1 million reads (150 bp) against the bacterial (pan-)genome, allowing a maximum error rate of 8%, i.e., at most $k = 12$ errors. For this dataset, BWA-MEM reports fewer co-optimal occurrences than Columba for almost 61% of the reads. In a pan-genome context, many reads indeed have many co-optimal alignments.

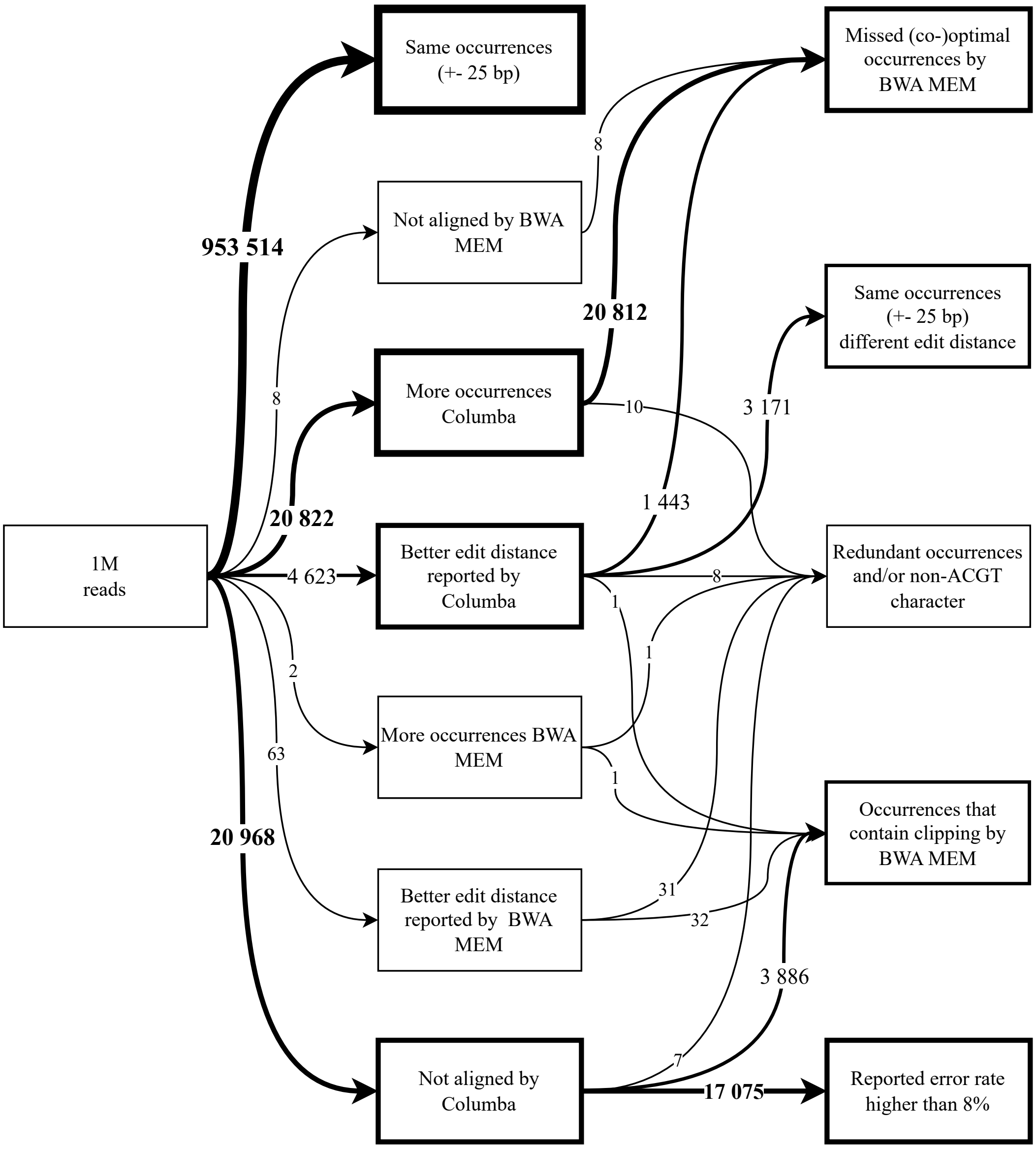

Figure 9: Comparison of the output between Columba and BWA-MEM2 for the alignment of 1 million reads (150 bp) against the human reference genome, allowing a maximum error rate of 8%.

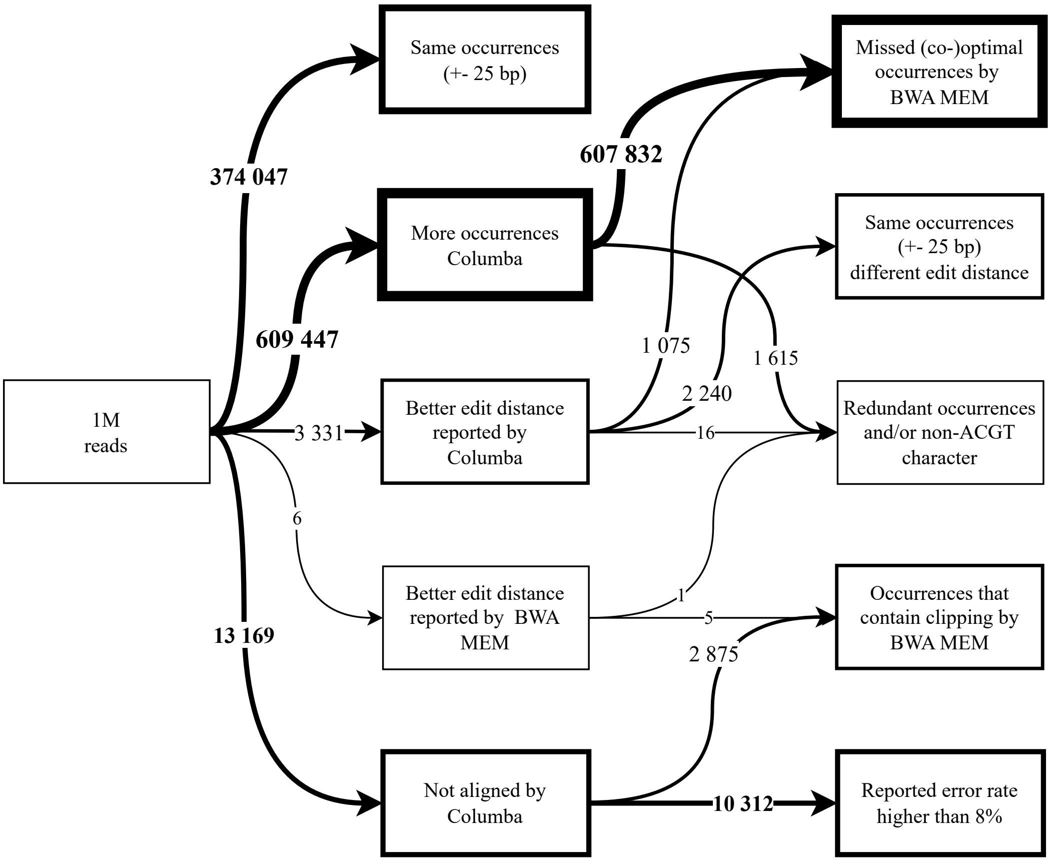

Figure 10: Comparison of the output between Columba and BWA-MEM for the alignment of 1 million reads (150 bp) against the bacterial pan-genome, allowing a maximum error rate of 8%.

5 Paired-end Alignment to the Human Reference Genome

Table 2: Runtime and peak memory usage of lossless alignment tools (Columba, Yara, BWA in aln mode, and Bowtie) at varying maximum error rate thresholds, and lossy alignment tools (BWA-MEM and Bowtie2 in normal and very sensitive (VS) modes), for aligning 1 000 000 pairs of Illumina reads (length 151 bp) from a larger WGS dataset to the human reference genome using a single thread. The alignment percentage of paired reads (reads mapped in proper pair) is noted alongside each runtime.

**Tool**	**Maximum Error Rate**				
	0%	2%	4%	6%	8%
**Lossless alignment tools**					
**Columba**	**44s** (45.4%)	**1m 57s** (82.7%)	**5m 01s** (89.8%)	**19m 54s** (92.9%)	**1h 49m 21s** (94.7%)
Yara	11m 04s (42.0%)	7m 14s (79.1%)	29m 36s (85.5%)	4h 32m 07s (88.2%)	1d 00h 55m 19s (89.8%)
BWA-aln	2m 42s (73.0%)	1h 22m 42s (89.8%)	7h 28m 21s (94.5%)	17h 01m 55s (94.6%)	1d 02h 50m 36s (94.6%)
Bowtie	**46s** (7.4%)	1h 11m 10s (11.2%)	not supported	not supported	not supported
**Lossy alignment tools**			**One Timing**		
BWA-MEM			16m 59s (99.5%)		
BWA-MEM2			9m 26s (99.5%)		
Bowtie2			13m 55s (84.5%)		
Bowtie2 (VS)			25m 45s (84.7%)		

Among lossless aligners, Columba consistently proves to be the fastest, completing the alignment in just 19m 54s at a 6% error rate, significantly outperforming Yara (4h 32m 07s) and BWA-aln (17h 01m 55s). Moreover, Columba's runtime remains competitive with lossy alternatives. The comparison of output across aligners is complicated by the distinct methods each tool uses to define "proper pair" mappings. Columba dynamically infers the mean fragment size and orientation, reporting pairs within six standard deviations of the detected mean fragment size. When multiple such pairs are possible, it selects those with the minimal combined edit distance.

The significantly higher alignment percentage observed for BWA-aln lower error rates can be attributed to its inclusion of alignments with clippings and its occasional allowance of errors in the mate, even when the maximum edit distance is strictly set to 0. Conversely, the significantly lower alignment percentage observed for Bowtie (both versions 1 and 2) can be attributed to its use of a fixed insert size, with default values of 250 and 500, respectively.

6 HLA Typing Experiment

6.1 Runtime Histogram for RNA Typing

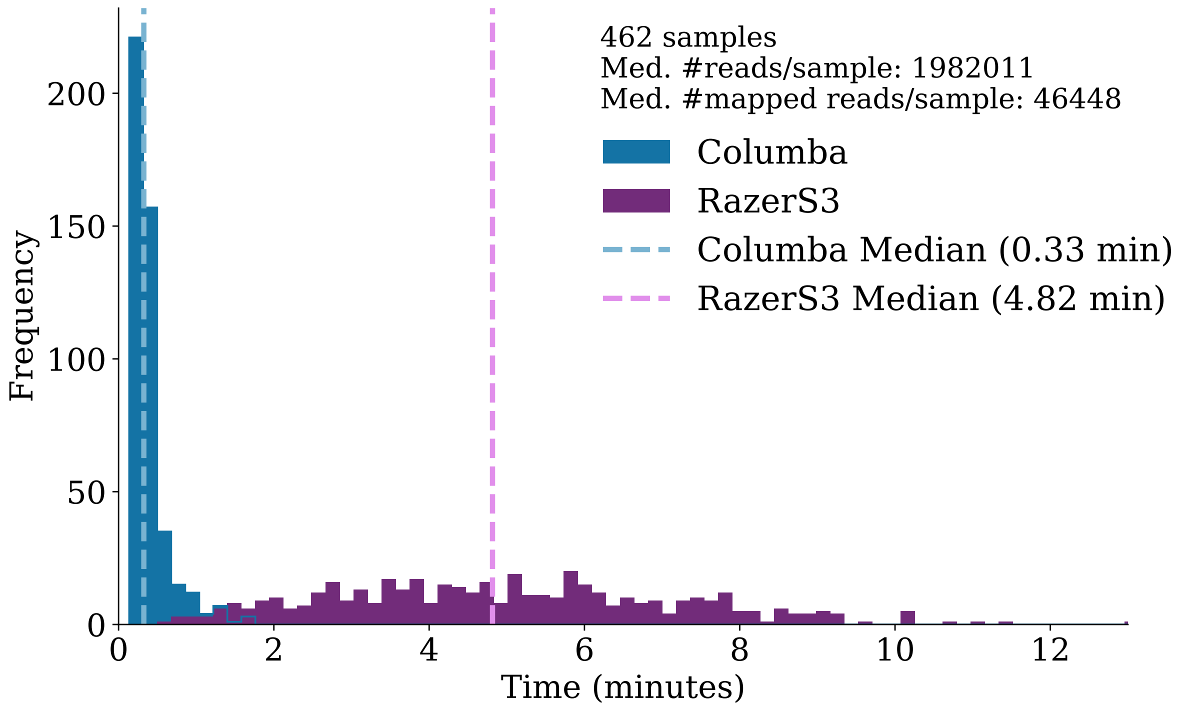

Figure 11: Comparison of the distribution of runtimes of the alignment phase of the Optitype pipeline with Columba and RazerS3 as the aligner for RNA sequences. Note that the figure is zoomed in on the range of 0 to 13 minutes, and that the distribution of RazerS3 has a tail that continues beyond this range.

6.2 Drop-in Nature of Columba in Optitype Script

```diff
 
     # Constants
     VERBOSE = ht.VERBOSE = bool(args.verbose)  # set verbosity setting in hlatyper too
-    COMMAND = "-i 97 -m 99999 --distance-range 0 -pa -tc %d -o %s %s %s"
+    COMMAND = "-I 97 -a best -nU -R -t %d  -o %s -r %s -f %s" # 97% identity, best mapping, no unmapped records, inputs are thread count, output file, reference base and reads sample
     ALLELE_HDF = os.path.join(this_dir, 'data/alleles.h5')
     MAPPING_REF = {'gen': os.path.join(this_dir, 'data/hla_reference_dna.fasta'),
                    'nuc': os.path.join(this_dir, 'data/hla_reference_rna.fasta')}
-    MAPPING_CMD = config.get("mapping", "razers3") + " " + COMMAND
+    MAPPING_CMD = config.get("mapping", "columba") + " " + COMMAND
     date = datetime.datetime.fromtimestamp(time.time()).strftime('%Y_%m_%d_%H_%M_%S')
     if args.prefix == None:
         prefix = date
@@ -273,10 +248,7 @@ def get_num_threads(configured_threads):
     if not os.path.exists(out_dir):
         os.makedirs(out_dir)
 
-    if PYSAM_AVAILABLE:
-        extension = 'bam'
-    else:
-        extension = 'sam'
+    extension = 'sam' # columba uses .sam output
 
     bam_paths = args.input if bam_input else [os.path.join(out_dir, ("%s_%i.%s" % (prefix, i+1, extension))) for i in range(len(args.input))]
 
@@ -290,14 +262,17 @@ def get_num_threads(configured_threads):
     # mapping fished file to reference
     if not bam_input:
         threads = get_num_threads(config.getint("mapping", "threads"))
+        base_path, _ = os.path.splitext(MAPPING_REF[ref_type])
+
         if VERBOSE:
           print("\nmapping with %s threads..." % threads)
         for (i, sample), outbam in zip(enumerate(args.input), bam_paths):
             if VERBOSE:
                 print("\n", ht.now(), "Mapping %s to %s reference..." % (os.path.basename(sample), ref_type.upper()))
 
+
             subprocess.call(MAPPING_CMD % (threads, outbam,
-                                           MAPPING_REF[ref_type], sample), shell=True)
+                                           base_path, sample), shell=True)
 
     # sam-to-hdf5
     table, features = ht.load_hdf(ALLELE_HDF, False, 'table', 'features')
```

Figure 12: Illustration of the changes required in the OptiType script to replace RazerS3 with Columba for HLA typing. This highlights that only minimal modifications are required to adapt existing workflows for Columba, showcasing its compatibility and flexibility.

7 Accession Numbers for the Bacterial Pan-genome

The accession numbers for the 6115 bacterial genomes are provided in the supplementary file `BacterialPanGenomeData.tsv`.

8 Multi-threaded Timings for the Bacterial Pan-genome

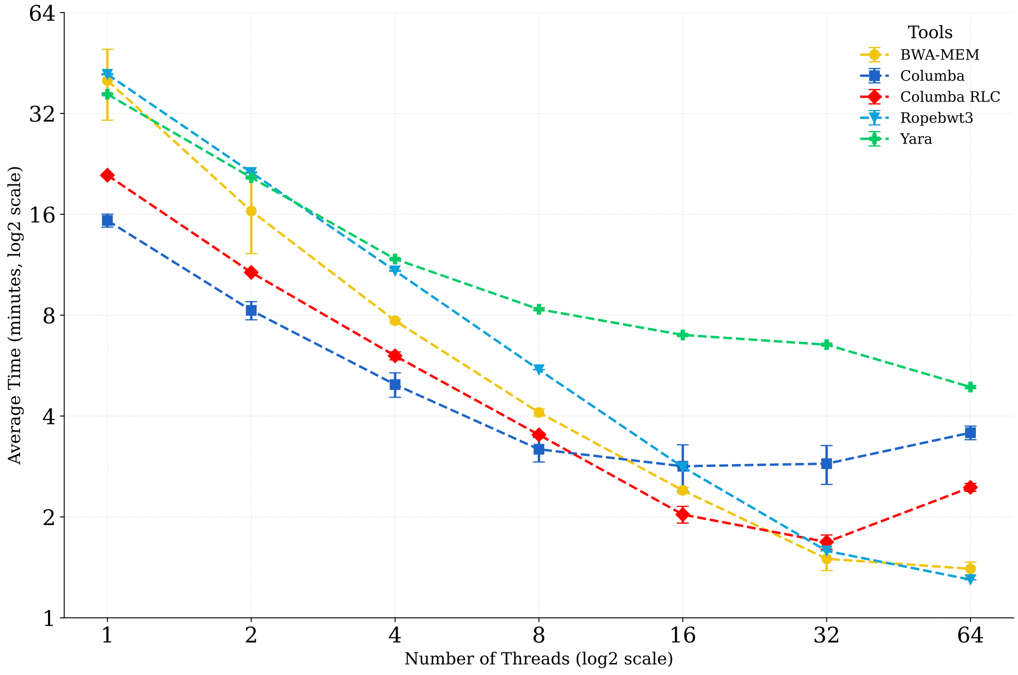

Figure 13: Multi-threaded timings for the benchmark where 1 million reads are aligned to the pangenome consisting of 6 115 bacterial genomes with various tools. The lossless tools (Yara and Columba) are configured with an allowed error rate of 5%. The lossless tools report significantly more occurrences than lossy aligners, leading to contention and throttling at higher thread counts due to disk write speed becoming a bottleneck.
